# The role of psychopharmacology and cognitive neuroscience in understanding the brain in the treatment of psychiatric disorders and neurological diseases for the benefit of society

**DOI:** 10.1177/02698811251397329

**Published:** 2025-12-01

**Authors:** Barbara J. Sahakian

**Affiliations:** 1 Department of Psychiatry, University of Cambridge, UK; 2 Behavioural and Clinical Neuroscience Institute, University of Cambridge, UK

**Keywords:** psychopharmacology, cognitive neuroscience, psychiatric disorders, neurological diseases

## Abstract

This perspectives piece reflects on some of the major scientific contributions in psychopharmacology, cognitive neuroscience, and public policy of Professor Barbara J. Sahakian, Commander of the Most Excellent Order of the British Empire (CBE). Her pioneering research has advanced the understanding of brain mechanisms, including neurotransmitter modulation, and psychological processes involved in cognition, emotion, and motivation, leading to novel treatments for disorders such as Alzheimer’s disease, attention deficit hyperactivity disorder, obsessive-compulsive disorder, and depression. She has also contributed to a better understanding of brain mechanisms underlying and psychological processes involved in these disorders. She has championed early detection of Alzheimer’s disease through neuropsychological tools, such as the Cambridge Neuropsychological Test Automated Battery (CANTAB) paired associates learning (PAL) test and contributed to identifying cognitive and neural changes in Huntington’s disease gene carriers. Beyond clinical research, Sahakian has influenced public health policy through initiatives such as the UK Government Foresight Project on Mental Capital and Wellbeing and the National Institute for Health and Care Excellence guidelines on gambling-related harms. She has also led efforts in neuroethics and public engagement, co-authoring accessible science books and participating in global forums. Recent research emphasises preventative psychiatry, including lifestyle interventions, such as diet, sleep, social connection, and lifelong learning as preventive strategies for cognitive decline and mental health problems. Through interdisciplinary collaborations and mentorship, Sahakian continues to inspire the next generation of scientists to pursue innovative research for societal benefit in neuropsychopharmacology and cognitive neuroscience.

## Introduction

I am very grateful to Professor Allan Young for the invitation to describe some of the research that has contributed to receiving the Commander of the Most Excellent Order of the British Empire for Services to Research in Human Cognitive Processes from His Majesty the King, Charles III, at Buckingham Palace in February 2025. In addition to this great honour, other highlights of my scientific, academic and clinical achievements include having been elected as a Fellow of the Academy of Medical Sciences (2004), and The British Academy (2017), and having received the Doctor of Science Degree (2015), which is the highest degree awarded by the University of Cambridge for distinguished research in science. In addition, I was extremely pleased to have received the Senior Investigator Award from the International College of Geriatric Psychoneuropharmacology (2010) and the Lifetime Achievement Award from the British Association for Psychopharmacology (2021), and the International College of Neuropsychopharmacology Pioneer Award (2026). A key reason for writing this piece is to inspire young psychopharmacologists and cognitive neuroscientists to join and contribute to these important fields.

Much of my work has focused on psychopharmacological studies to understand the role of neurotransmitters, including dopamine, noradrenaline, and serotonin, in the modulation of components of cognition, emotion, and motivation in health and in psychiatric disorders and neurological diseases. The aim of this work was not only to understand the mechanisms of action in the healthy brain, but also to develop novel, more effective treatments for cognitive dysfunction in patients.

In regard to success with the development of novel treatments, I was one of the first researchers to suggest that attentional dysfunction in Alzheimer’s disease (AD) could be ameliorated using pharmacotherapy, such as cholinesterase inhibitors. My earlier observations indicated that these drugs would enhance signal-to-noise ratio in the forebrain cholinergic system, which modulates attention (Sahakian et al., 1993). Our randomised, double-blind, placebo-controlled trial for cholinesterase inhibitors as a treatment for cognitive problems in mild to moderate AD was published in The Lancet (Eagger et al., 1991). Cholinesterase inhibitors (e.g. Aricept and Donepezil) are now widely used and approved by regulatory bodies globally and by the National Institute for Health and Care Excellence (NICE) as a treatment for AD, and their use is on the increase.

I have also investigated the neurochemical modulation of impulsive and compulsive behaviour in neuropsychiatric disorders, including attention deficit hyperactivity disorder (ADHD) (Pironti et al., 2014; Shen et al., 2020) and obsessive-compulsive disorder (Chamberlain et al., 2008). My recent articles on ADHD (Del Campo et al., 2013; Pironti et al., 2014; Shen et al., 2020) have implications for determining the underlying neural mechanisms involved, and also clarifying the action of the drug methylphenidate (Ritalin) on cognition. In a series of papers, my PhD students and I were able to discover that a neural circuit including the right inferior frontal gyrus was responsible for response inhibition (Aron et al., 2003). Our studies have questioned the previously accepted view that major abnormalities in dopamine function are the major cause of ADHD (Del Campo et al., 2013; Pironti et al., 2014; Shen et al., 2020). These findings indicated that the main cause of the disorder may instead be associated with structural differences in grey matter in the brain. Recently, we have also shown that emotion dysregulation was associated with ADHD symptoms, and this persisted after controlling for the cognitive and motivational deficits (Hou et al., 2024). Emotion dysregulation mediated the association between a smaller surface area of the right pars orbitalis and greater ADHD symptoms at 1-year follow-up, indicating an emotion pathway for ADHD. Our study findings suggested that emotion dysregulation is a core symptom and route to ADHD, which may not respond to the current pharmacological treatments for ADHD.

In the course of conducting my research on ‘cognitive enhancing’ drugs, my students and I found that they not only improved cognition in people with neuropsychiatric disorders, but also in healthy volunteers (Chamberlain et al., 2006; Turner et al., 2003), including in sleep-deprived doctors (Sugden et al., 2012). This work has relevance to the use of such drugs as methylphenidate and modafinil by healthy people to improve cognitive performance, increase task-related motivation, reduce sleepiness, and counteract the effects of jet lag (Sahakian et al., 2015). Our findings and other evidence suggest that these drugs enhance cognitive effort (Müller et al., 2013).

In terms of compulsivity, much of the research has focused on the misuse of substances, including cocaine and cannabis, and on compulsive behaviours as seen in obsessive-compulsive disorder and gambling. Our key article published in the Journal of Psychopharmacology showed cognitive impairments in visual and episodic memory in young adults with cannabis use disorder (Selamoglu et al., 2021). In addition, executive dysfunction was associated with the age of onset in these daily cannabis users. We have also shown that adolescent and adult dependent cannabis users had significantly higher apathy and anhedonia compared to non-dependent users and controls (Skumlien et al., 2021).

Being on the Committee for the NICE guidelines on Gambling-Related Harms: Identification, Assessment, and Management (2025) has been rewarding in identifying future research needed in this area for more effective treatments, including pharmacological ones (https://www.nice.org.uk/guidance/ng248). All these disorders of compulsivity lead to a narrowing of goal-directed behaviour and a focus on habitual behaviour, involving brain areas such as the dorsal striatum, particularly the putamen.

## Early detection of Alzheimer’s disease with neuropsychological measures

Based on theoretical and experimental neuroscience and neuropsychological findings, including the site of early neuropathological changes and the functional role of the hippocampal formation, I was one of the first to show that new learning and memory for the location of objects in space is affected at the earliest stages of AD. I published a seminal first-authored paper in the journal Brain in 1988, demonstrating the sensitivity of a Cambridge Neuropsychological Test Automated Battery Paired Associates Learning test (CANTAB PAL) to AD (Sahakian et al., 1988). This paper contrasted the marked deficits seen in visuospatial learning and memory in patients with mild AD with the relative sparing of these processes in patients with Parkinson’s disease. CANTAB PAL is now used in what the Medical Research Council – United Kingdom Research and Innovation (MRC-UKRI) have termed ‘the world’s most in-depth study to detect early signs of Alzheimer’s Disease’ (Koychev et al., 2019). In March 2021, the PAL task was added to the Brain Health Registry, an online, remote research study run by the University of California, San Francisco that collects longitudinal, unsupervised data related to cognitive ageing and dementias in adults (*N* > 100,000) (Ashford et al., 2024). In terms of translation from the laboratory into the clinic, CANTAB PAL now also runs on iPads as CANTAB mobile for use in the clinic for the early detection of amnestic mild cognitive impairment (MCI) in the elderly. CANTAB is used in over 1000 hospitals, universities, and institutes in over 100 countries. In a 2011 study (de Rover et al., 2011), my colleagues and I demonstrated that during the PAL task, the hippocampi of MCI patients were activated significantly more than controls at lower levels of difficulty during the test, and significantly less at higher levels of difficulty, thus providing neural validation for the CANTAB PAL test in the early detection of AD. The earliest neuropathology in AD is seen in the entorhinal cortex and hippocampus. I was a pioneeri proponent of the importance of early detection of AD in order to improve functional outcome and quality of life by pharmacological symptomatic treatments through training/compensatory techniques and through advanced planning of life goals and financial and clinical management. I also suggested that early detection of amnestic MCI was important because of the ongoing development of neuroprotective agents that may slow or halt the disease process, such as the FDA-approved lecanemab.

More recent work has focused on Huntington’s disease (HD), where, in collaboration with Professor Sarah Tabrizi at University College London, we have been examining cognition, neuroimaging, and biofluids in far from clinical motor onset young adults with the HD gene expansion (HDGE). This longitudinal study has demonstrated some subtle early deficits in cognition, volumetrics, functional connectivity, and biofluids (Scahill et al., 2020, 2025). Langley et al. (2021) demonstrated early changes in the fronto-striatal circuitry, which were associated with poorer cognitive flexibility in HDGE, 24 years from predicted onset. Cognitive Flexibility is an important cognitive process necessary for learning in novel situations or under uncertainty and in adapting to a changing environment (Lee et al., 2024; Tong et al., in submission). We hope this HDGE group can participate in future studies that are aimed at halting or slowing disease progression prior to the occurrence of motor symptoms and cognitive impairments affecting daily living.

## Understanding core cognitive changes in depression

Depression is frequently a chronic, relapsing condition that is responsible for an enormous personal cost to patients and their families, but also a financial cost to the government due to the fact that depression is very common and many days of work are lost. Innovative research, by me and my students, has demonstrated that there are cognitive impairments in depression, which may, in a proportion of people, still remain following treatment and symptom improvement. Some of these impairments are ‘cold’ or non-emotional, such as planning and problem solving, and others are ‘hot’ and involve risky decision making or tasks in which there is a conflict between reward or gain and punishment or loss (Elliott et al., 1996, 1997; Roiser et al., 2012). My research has focused on what I regard as the essence of the neurocognitive problem in depression: an attentional bias to negative stimuli in the environment and an abnormal response to negative feedback (Murphy et al., 1999). Using functional magnetic resonance imaging (fMRI), colleagues and I demonstrated the neural correlates of these psychological processes. Significantly, for emotional regulation under stressful circumstances, such as negative feedback, healthy, never depressed individuals exert top-down control by the prefrontal cortex over the amygdala, which is deactivated (Tavares et al., 2008). Crucially, depressed individuals are unable to do so. This work on cognition, depression, and implications for treatment have been highlighted in reviews (Clark et al., 2009; Roiser et al., 2012).

Recently, with Professor Gitte Moos Knudsen at the University of Copenhagen, we have been examining the effects of 3 weeks of escitalopram on cognition, neuroimaging, and neural plasticity in healthy volunteers. We used computational modelling to demonstrate reduced reinforcement sensitivity following escitalopram administration, with cold cognition and other forms of hot cognition remaining intact (Langley et al., 2023). This was an important finding as it may relate to the blunting of emotion regarded as a side-effect of selective serotonin reuptake inhibitor treatment, although this blunting may be part of the initial therapeutic process, especially under high levels of anxiety. This was followed up by a neuroimaging study demonstrating impaired punishment learning, which was associated with reduced activity in the intraparietal sulcus in the escitalopram group (Langley et al., 2025). Another of our studies examining an emotional faces paradigm during fMRI found that escitalopram administration impacts cortical responses in the frontal and occipital regions in response to emotional faces (Armand et al., 2024). Importantly, in a subset that underwent Positron Emission Tomography with the [11C] UCB- ligand,a marker of neural plasticity, we found that there was a significant positive correlation between neural plasticity and time on escitalopram, which may explain the delay in the symptom improvement of patients taking escitalopram (Johansen et al., 2023).

## Policy and public engagement

I have been involved in a number of policy publications, which have influenced government policy on public health. I have also been very active in engaging the public in neuroscience and its importance for mental health. I was the neuroscience lead on the UK Government Foresight Project on Mental Capital and Wellbeing (Beddington et al., 2008), which has had an enormous impact on government policy, including ‘Research Changes Lives’: Medical Research Council Strategic Plan 2009–2014. Furthermore, in terms of national and global impact, the concepts advanced, including wellbeing and resilience, have been incorporated into many aspects of government policy and public health. In addition, I was a co-author and member of the Scientific Advisory Board on the Grand Challenges in Global Mental Health (Collins et al., 2011). I was a speaker on cognitive enhancement and mental wellbeing at the World Economic Forum (WEF) 2014 in Davos and was later appointed to two committees for the WEF (Global Agenda Council on Brain Research and Future of Neurotechnologies and Brain Science). I was on the Planning Committee for enabling discovery, development, and translation of treatments for cognitive dysfunction in depression for the National Academies of Sciences, Engineering, and Medicine Forum on Neuroscience and Nervous System Disorders (USA). Recently, I have been an expert witness for the Science and Technology Committee at the House of Lords on the topic of Ageing: Science, Technology and Healthy Living (2020) and the academic representative on the Committee for the NICE guidelines on harmful gambling (2022–2024).

My scientific work has been pioneering in the development of the field of neuroethics and highlighted the importance of the engagement of the public in science. In 2006, I became a founder member in the Executive Committee of the International Neuroethics Society (http://www.neuroethicssociety.org), funded by the Dana Foundation, and was President from 2014 to 2016. I am committed to teaching neuroethics and engagement of the public in science to young neuroscientists, and have advocated this through my publications, including the Oxford Handbook of Neuroethics (Illes and Sahakian, 2011), the popular science books Bad Moves: How decision making goes wrong and the ethics of smart drugs (Sahakian and LaBuzetta, 2013) and Sex, Lies, and Brain Scans. How fMRI reveals what really goes on in our minds (Sahakian and Gottwald, 2016). Both popular books were reviewed positively in Nature and Sex, Lies & Brain Scans received the British Psychological Society Popular Science Book Award. I contributed to the Royal Society’s ‘Brain Waves’ project. I have also participated in many public science festivals, radio, TV, newspapers, and magazine interviews, including a BBC 1 documentary (2021), BBC Two Newsnight and regular interviews on the BBC Radio 4 Today Programme. According to Meltwater (the University of Cambridge’s media monitoring service), ‘Barbara Sahakian’ was mentioned in 4053 media articles and blogs between January–July 2022. I have also published several articles in The Conversation, a media outlet for the public with evidence-based articles, reaching over 4 million readers. For this work, I received The Conversation’s Sir Paul Curran Award in 2023.

My current research has moved towards preventative psychiatry through the prevention of cognitive impairments and mental health problems by considering lifestyle factors that can improve brain health and wellbeing. Many of the published studies are presented in a new co-authored book with Dr Christelle Langley, entitled *Brain Boost: Healthy Habits for a Happier Life* (Sahakian and Langley, 2025).

The book, which was written for the general public, provides an evidence-based guide to improve both our physical and mental health by adopting healthy lifestyle factors and maintaining them throughout life. Brain Boost also provides an excellent evidence-based guide for medical general practitioners for social prescribing and will make it easier to explain to patients both why they should be changing their lifestyle to improve their physical and mental health, and also how they can do this. The book goes into detail a number of factors, including on how our diet (Kang et al., 2022; Zhang et al., 2024), sleep (Li et al., 2022), and social support systems (Shen et al., 2022, 2023) are all key to improving brain health, cognition, and wellbeing. For example, a diet with high cereal and low caffeine was associated with better cognitive performance, body mass index, and other metabolic measures (Kang et al., 2022). My collaborative research studies have also shown that in middle to older aged adults, getting 7–8 hours of sleep per night consistently was associated with better cognition, brain health, and mental health (Li et al., 2022).

My work has had a particular focus on social isolation and loneliness, as the World Health Organisation (2023) has reported that one in four older adults is socially isolated and 5%–15% of adolescents feel lonely. Indeed, we have found that social isolation and loneliness can detract from our brain health and wellbeing. In fact, in this older age group, social isolation can actually increase the risk of dementia by 26% (Shen et al., 2022). Our recent proteomic study highlighted a number of proteins that appear to play a key role in the relationship between loneliness and poorer health, including heart disease, stroke, susceptibility to infection, and mortality (Shen et al., 2025). In addition, we found that having a healthy lifestyle can reduce the risk of depression by 57% compared to having an unhealthy lifestyle and may even help mitigate the effects of genetic risk factors (Zhao et al., 2023). Another factor is lifelong learning, including cognitive games, which can improve cognition in healthy individuals (Savulich et al., 2019). In addition, these games improved cognitive performance and functioning in people with neurological diseases (Savulich et al., 2017; Studer et al., 2021) and psychiatric disorders (Sahakian et al., 2015). Furthermore, lifelong learning can build cognitive reserve and foster resilience, which can also improve functioning and lead to better outcomes in people with neurological diseases and psychiatric disorders (Barnett et al., 2006; Salmond et al., 2006; Southwick and Charney, 2018).

On the other end of the age spectrum, having a friendship group of approximately 5 close friends in late childhood was associated with beneficial effects on cognition, brain health, and mental health (Shen et al., 2023). In another study, we found that reading for pleasure early in childhood is associated with better brain structure, cognition, educational attainment, and mental health in adolescence (Sun et al., 2024). These adolescents also had better sleep and less screen time, including TV, cell phone, and tablets. This demonstrates how adopting good strategies and habits early in life can have a major positive impact on development and functioning later in life.

As artificial intelligence (AI) has become increasingly important in science and medicine, some of our studies have utilised machine learning to understand the effects of biological sex and environment on the human brain (Zhang et al., 2021). We have also used theory of mind in attempts to improve AI function in driverless cars and in interactions with humans in the workplace (Cuzzolin et al., 2020; Langley et al., 2022). Understanding hot and cold cognitive processes is not only important for human decision making, especially under conditions of risk or uncertainty (Lawrence et al., 2008), but also for algorithms used in financial investment decision making (Buczynski et al., 2021).

Finally, I am very grateful to my many outstanding collaborators, postdoctoral researchers, and PhD students, who have contributed to this research. I hope that this article will encourage many young scientists to pursue careers in these research topics and in these different areas of endeavour (Sahakian, 2014). Hopefully, this will lead to answering many of the challenging questions in neuroscience and to the prevention of some mental health disorders, as well as to the development of novel, more effective treatments for those with psychiatric disorders and neurological diseases to ensure a better quality of life and improved wellbeing.

For you, PhD students, Postdoctoral Researchers, and Early Career Scientists and Academics, I hope that you will join in these important areas of research and have confidence in your ability for innovative thinking and creative research to contribute to ensuring a healthier and happier society. I also hope that the government and Charities will increase their funding to support the career development of young scientists in these key areas of psychopharmacology, cognitive neuroscience, and mental health, to ensure better brain health and wellbeing for everyone.
